# Reversible severe tricuspid regurgitation associated with Graves’ disease: A case report

**DOI:** 10.1097/MD.0000000000028432

**Published:** 2021-12-23

**Authors:** Ja-Yeon Lee, Sun Hwa Lee, Won Ho Kim

**Affiliations:** Division of Cardiology, Department of Internal Medicine, Jeonbuk National University Medical School, Research Institute of Clinical Medicine of Jeonbuk National University-Biomedical Research Institute of Jeonbuk National University Hospital, Jeonju, Republic of Korea.

**Keywords:** Graves’ disease, heart failure, tricuspid regurgitation

## Abstract

**Rationale:**

Graves’ disease is the most common cause of thyrotoxicosis. Cardiovascular signs and symptoms are frequent in patients with thyrotoxicosis and right heart failure with severe tricuspid regurgitation (TR) is a rare manifestation of hyperthyroidism.

**Patient concerns:**

A 41-year-old woman with a history of Graves’ disease presented to the emergency department with worsening generalized edema and dyspnea for a month.

**Diagnosis:**

The laboratory test results revealed suppressed thyroid-stimulating hormone (TSH), elevated levels of free thyroxine and anti-TSH receptor antibody, and negative anti-thyroid peroxidase and anti-thyroglobulin antibodies. Transthoracic echocardiography showed severe TR associated with incomplete coaptation of tricuspid valve due to dilated right ventricle (RV), moderate resting pulmonary hypertension, and preserved biventricular systolic function.

**Interventions and outcomes:**

After 6 months of antithyroid treatment, her thyroid function was restored euthyroid state and she was fully recovered from right heart failure. Follow-up echocardiography showed complete disappearance of severe TR and pulmonary hypertension and normalization of RV dimension.

**Lessons:**

Severe TR can be rarely associated with thyrotoxicosis, but this is reversible and can be completely recovered with normalization of thyroid function.

## 1 Introduction

Graves’ disease is the most common cause of thyrotoxicosis and can cause cardiovascular complications. Thyroid hormones are closely related to the function and the structure of the heart. Excess thyroid hormones induce significant cardiovascular hemodynamic changes.^[1]^ Arterial hypertension and atrial fibrillation (AF) are the most common complications. Heart failure (HF) and valvular dysfunction have also been rarely reported.^[2]^ Right heart failure (RHF) is a more uncommon presentation than left-sided HF in patients with hyperthyroidism.^[3]^ Here, we present a case of uncontrolled thyrotoxicosis-related severe TR with RHF, fully recovered with the attainment of euthyroid state.

## 2 Case presentation

A 41-year-old woman presented to the emergency department with worsening generalized edema and dyspnea for a month. She was diagnosed with Graves’ disease at a primary clinic 18 months ago. Methimazole was prescribed for 10 months and has been discontinued for 8 months after restoration to normal thyroid function. However, Graves’ disease relapsed and methimazole was resumed since last month. Despite treatment, worsening pedal edema and dyspnea brought her to the emergency room.

**Figure 1:**
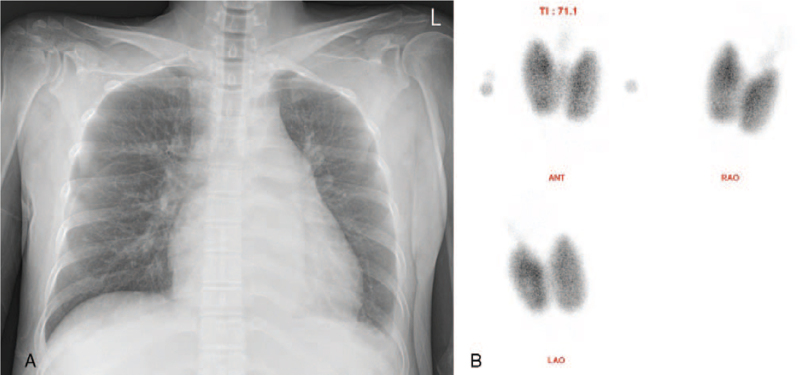
A, A chest radiograph showing mild cardiomegaly and blunted costophrenic angle. B, Technetium-99m thyroid scan demonstrating diffuse bilateral enlargement of thyroid gland with intense homogeneous radiotracer uptake suggesting Graves’ disease.

**Figure 2:**
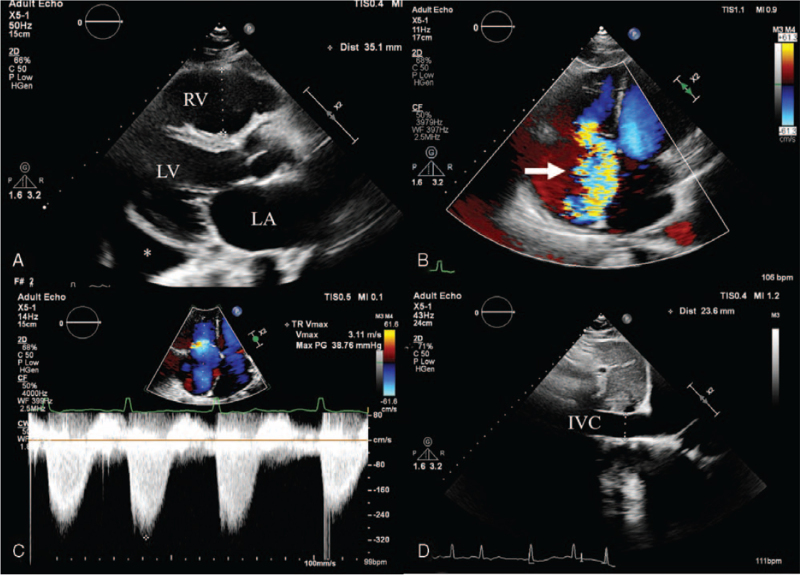
Transthoracic echocardiography at the time of diagnosis of severe tricuspid regurgitation (TR) showing (A) dilated right ventricle, (B) severe TR (arrow) on a color Doppler image, and (C–D) moderately increased pulmonary artery systolic pressure of 59 mmHg with inferior vena cava plethora. IVC = inferior vena cava, LA = left atrium, LV = left ventricle, RV = right ventricle; asterisk = pleural effusion.

**Figure 3:**
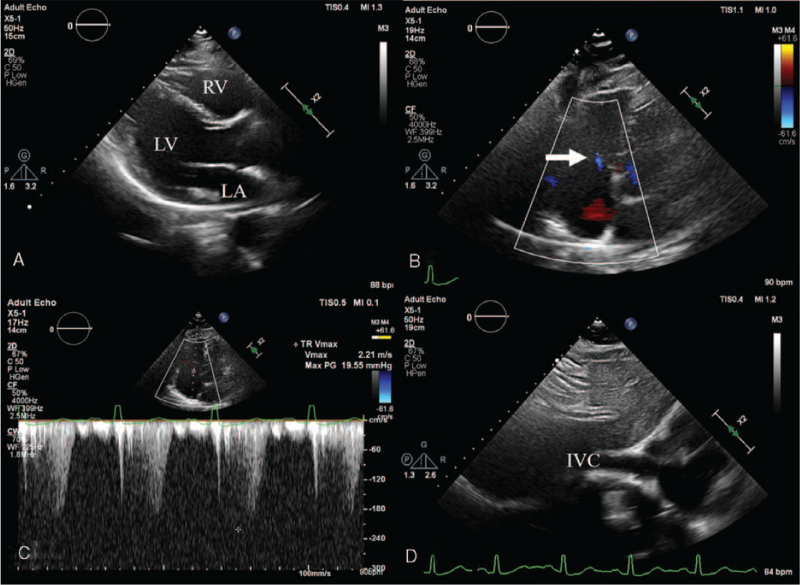
Six-month follow-up echocardiographic images after attainment of euthyroid state showing (A) normalized size of the right ventricle, (B) near complete resolution of tricuspid regurgitation (arrow), and (C–D) normalization of pulmonary artery systolic pressure and inferior vena cava diameter. IVC = inferior vena cava, LA = left atrium, LV = left ventricle, RV = right ventricle.

At presentation, her blood pressure was 116/73 mmHg, heart rate 91 beats/min, respiratory rate 20/min, and body temperature 36.2 °C. Physical examination revealed exophthalmos, a diffuse goiter, irregular heartbeats with grade 3/6 systolic murmur at the left lower sternal border, and grade 3 pitting edema at both lower legs. Chest radiographs showed cardiomegaly with a cardiothoracic ratio of 0.56 (Fig. 1A) and small amount of fluid shift on bilateral decubitus views. An electrocardiogram (ECG) demonstrated AF at a rate of 87 bpm. Laboratory test revealed suppressed thyroid-stimulating hormone (TSH, 0.007 μIU/mL), elevated levels of free thyroxine (T4, 37.63 pmol/L) and anti-TSH receptor antibody (27.12 IU/L), and negative anti-thyroid peroxidase (anti-TPO Ab, 21.23 IU/mL) and anti-thyroglobulin (anti-TG Ab, 14.70 IU/mL) antibodies. The levels of liver enzymes were mildly increased (aspartate aminotransferase, 50 IU/L; alanine aminotransferase, 45 IU/L) and N-terminal pro-brain natriuretic peptide was also elevated to 1007 pg/mL. Technetium-99m thyroid scan showed bilateral diffuse enlargement of thyroid gland and intense homogeneous radiotracer uptake, consistent with Graves’ disease (Fig. 1B). Transthoracic echocardiography was performed to assess the etiology of HF and demonstrated severe tricuspid regurgitation (TR) associated with incomplete systolic coaptation of tricuspid valve due to dilated right ventricle (RV) (Fig. 2A, B), moderate resting pulmonary hypertension with pulmonary artery systolic pressure of 59 mmHg (Fig. 2C, D), normal left ventricular (LV) dimension, normal LV systolic function with ejection fraction of 59%, borderline elevation of LV end-diastolic pressure with *E*/*E*′ ratio of 12.5, and preserved RV systolic function. She underwent enhanced chest computed tomography to identify the presence of pulmonary thromboembolism as a cause of RHF, which showed bilateral pleural effusion, minimal ascites, and no evidence of pulmonary embolism. Generalized edema and dyspnea were gradually improved with administration of furosemide, propranolol, and methimazole. After 6 months, euthyroid state was restored. In addition, follow-up ECG showed spontaneous conversion to normal sinus rhythm. Echocardiography revealed scanty TR with normalization of RV dimension and pulmonary artery systolic pressure of 27 mmHg (Fig. 3). Diuretics and beta-blocker were discontinued and methimazole was gradually tapered. She has been doing well and thyroid hormone levels have been maintained within the normal range for 6 months.

## 3 Discussion

Graves’ disease is an autoimmune disorder characterized by hyperthyroidism or thyrotoxicosis, a state of excessive thyroid hormones. Thyroid hormones have considerable effects on the energy metabolism of the heart, regulating cardiac function, and cardiovascular hemodynamics. In thyrotoxicosis, overproduced thyroid hormones lead to hemodynamic changes of the cardiovascular system.^[1]^

Graves’ disease can cause thyrotoxic heart disease if left untreated or poorly controlled. Systemic arterial hypertension, tachyarrhythmias including AF and supraventricular premature complexes (SPC), and pulmonary hypertension are well recognized cardiac complications.^[1]^ Left ventricular failure, isolated RHF, and valvular dysfunction rarely complicate even in young patients without pre-existing heart disease.^[4]^ A prospective study by Tsymbaliuk et al^[5]^ showed that 75 out of 1194 patients with Graves’ disease (6.3%) presented with cardiovascular symptoms. Systemic hypertension was the most common manifestation (95%), followed by AF, SPC, HF, and pulmonary hypertension. In 2% to 25% of patients, these problems improved after antithyroid treatment.

In general, the etiology of significant TR is secondary in ≥90% of cases and is caused by dilation of right heart due to pressure or volume overload resulted from left-sided valve disease or left ventricular failure.^[6]^ The mechanism of thyrotoxicosis-associated TR is not fully understood and is thought to be related to increased total blood volume and resting heart rate, changes in myocardial contractility, and decreased peripheral vascular resistance.^[7]^ The resultant hyperdynamic circulation may alter the function and mechanics of thin-walled RV, leading to dilatation of the right heart, which contributes to further aggravation of TR.^[8]^

Since severe TR is associated with poor outcomes and worsening HF, medical or surgical treatment is indicated in patients with severe symptomatic TR. Because of limited knowledge about natural history and outcomes of medical or surgical intervention for TR, the treatment decision making is often challenging. The etiology of TR is a major determinant of the treatment strategy. For secondary TR, treatment focuses on appropriate medical therapy for pressure or volume overload and surgical treatment is performed only for selected patients.^[9]^

This patient with uncontrolled Graves’ disease presented with cardiovascular symptoms including biventricular HF and AF. Among them, RHF associated with severe TR was the dominant problem. Because the patient was a young woman, we also took into consideration the possibility that an undiagnosed underlying condition, such as primary pulmonary arterial hypertension, was exacerbated by thyrotoxicosis. However, in this patient, restoring to the euthyroid state led to complete resolution of severe TR and signs of HF. It is thought to be related to the normalization of overactivated circulatory dynamics, resulting in reduced pressure or volume overload on the right heart.

In conclusion, thyrotoxicosis is a rare but reversible cause of symptomatic significant TR. Clinicians should keep hyperthyroidism in mind as one of the differential diagnoses of symptomatic TR.

## Author contributions

**Conceptualization:** Sun Hwa Lee, Won Ho Kim.

**Data curation:** Ja-Yeon Lee, Sun Hwa Lee.

**Supervision:** Won Ho Kim.

**Writing – original draft:** Ja-Yeon Lee.

**Writing – review & editing:** Sun Hwa Lee, Won Ho Kim.

## References

[1] Osuna PM Udovcic M Sharma MD. Hyperthyroidism and the heart. Methodist Debakey Cardiovasc J 2017;13:60–3. 28740583 10.14797/mdcj-13-2-60 PMC5512680

[2] Witczak JK Ubaysekara N Ravindran R Rice S Yousef Z Premawardhana LD. Significant cardiac disease complicating Graves’ disease in previously healthy young adults. Endocrinol Diabetes Metab Case Rep 2020;2020:19–0132. 10.1530/EDM-19-0132 PMC6993248 31967967

[3] Bonou M Lampropoulos KM Andriopoulou M Kotsas D Lakoumentas J Barbetseas J. Severe tricuspid regurgitation and isolated right heart failure due to thyrotoxicosis. Indian Heart J 2012;64:600–2. 23253416 10.1016/j.ihj.2012.09.005 PMC3861025

[4] Cappola AR Desai AS Medici M. Thyroid and cardiovascular disease: research agenda for enhancing knowledge, prevention, and treatment. Circulation 2019;139:2892–909. 31081673 10.1161/CIRCULATIONAHA.118.036859 PMC6851449

[5] Tsymbaliuk I Unukovych D Shvets N Dinets A. Cardiovascular complications secondary to Graves’ disease: a prospective study from Ukraine. PLoS One 2015;10:e0122388. 25803030 10.1371/journal.pone.0122388 PMC4372210

[6] Topilsky Y Maltais S Medina Inojosa J. Burden of tricuspid regurgitation in patients diagnosed in the community setting. JACC Cardiovasc Imaging 2019;12:433–42. 30121261 10.1016/j.jcmg.2018.06.014

[7] Pierre K Gadde S Omar B Awan GM Malozzi C. Thyrotoxic valvulopathy: case report and review of the literature. Cardiol Res 2017;8:134–8. 28725332 10.14740/cr564w PMC5505299

[8] Rana BS Robinson S Francis R. Tricuspid regurgitation and the right ventricle in risk stratification and timing of intervention. Echo Res Pract 2019;6:R25–39. 30763278 10.1530/ERP-18-0051 PMC6410762

[9] Otto CM Nishimura RA Bonow RO. 2020 ACC/AHA guideline for the management of patients with valvular heart disease: a report of the American College of Cardiology/American Heart Association Joint Committee on Clinical Practice Guidelines. J Am Coll Cardiol 2021;77:450–500. 33342587 10.1016/j.jacc.2020.11.035

